# Comparative evaluation of cognitive behavioural therapy versus standard treatment in temporomandibular disorders: A systematic review

**DOI:** 10.1111/joor.13792

**Published:** 2024-07-23

**Authors:** Sahana Shivakumar, Nishath Sayed Abdul, Bhuvan Jyoti, Veena Kalburgi, Marco Cicciù, Giuseppe Minervini

**Affiliations:** ^1^ Public Health Dentistry Peoples College of Dental Sciences and Research Centre Peoples University Bhopal Bhopal India; ^2^ Faculty of Oral Pathology, Department of OMFS and Diagnostic Sciences Riyadh Elm University Riyadh Riyadh Saudi Arabia; ^3^ Dental Surgeon and Consultant Oral Medicine and Radiology Ranchi Institute of Neuropsychiatry and Allied Sciences (RINPAS) Ranchi India; ^4^ Periodontics Peoples College of Dental Sciences and Research Centre Peoples University Bhopal Bhopal India; ^5^ Department of Biomedical and Surgical and Biomedical Sciences Catania University Catania Italy; ^6^ Saveetha Dental College and Hospitals, Saveetha Institute of Medical and Technical Sciences (SIMATS) Saveetha University Chennai India; ^7^ Multidisciplinary Department of Medical‐Surgical and Dental Specialties University of Campania "Luigi Vanvitelli" Naples Italy

**Keywords:** cognitive‐behavioural therapy, pain, temporomandibular disorders

## Abstract

**Background:**

Temporomandibular disorders (TMDs) are musculoskeletal and neuromuscular conditions affecting the temporomandibular joint and associated structures. Cognitive‐behavioural therapy (CBT) has emerged as a potential intervention for TMDs, but its comparative effectiveness against standard treatments remains unclear. This systematic review aimed to evaluate and compare the efficacy of CBT versus standard treatment interventions in managing TMDs.

**Methods:**

A comprehensive search was conducted across multiple databases using MeSH keywords and Boolean operators. Inclusion criteria encompassed clinical trials comparing CBT/CBT in combination with standard treatment interventions or a control group in individuals with TMDs. The primary outcome measured was pain. Secondary outcomes such as disability, depression and jaw function were also looked into. Two reviewers independently assessed for the eligibility of the articles and conducted data extraction. Quality assessments were performed using RoB 2.0 for randomised clinical trials.

**Results:**

The initial search identified 623 articles, of which a total of eight clinical studies met the inclusion criteria and were included in the systematic review. Seven out of eight studies demonstrated improvements in outcomes related to TMD. Pain was significantly decreased in studies that showed a positive outcome. Jaw function, quality of life and psychological well‐being were superior among individuals receiving CBT alone or in combination with conventional modalities, as well as hypnotic therapy coupled with CBT‐based interventions. The quality of studies assessed showed all articles to be of good quality as per RoB‐2 evaluation.

**Conclusion:**

This systematic review highlights the potential benefits of CBT in managing TMDs, suggesting its effectiveness in improving pain outcomes and enhancing overall well‐being. The findings indicate that CBT may be a valuable adjunct or alternative to standard treatment interventions for individuals with TMDs. However, further research with larger sample sizes and standardised outcome measures is warranted to establish definitive conclusions regarding the comparative efficacy of CBT versus standard treatments in TMD management.

## Graphical abstract

Graphical abstract of this systematic review.

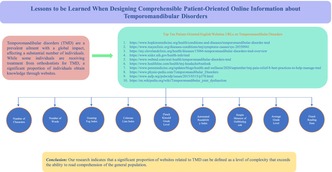

## 1 INTRODUCTION

Temporomandibular disorders (TMDs) are a group of musculoskeletal and neuromuscular conditions that affect the Temporomandibular joint (TMJ), facial muscles and associated structures. (Table 1) TMDs are characterised by symptoms such as pain, jaw dysfunction, clicking or popping sounds and limited mouth opening.^1^ These disorders can significantly impact an individual's quality of life, leading to difficulties in eating, speaking and performing daily activities. The prevalence of TMD pain in the general adult population is estimated to be between 10% and 15%, predominantly affecting females. Among adolescents, the prevalence rate is approximately 27%.^2^, ^3^ Different conditions associated with TMDs have been known to negatively impact the overall OHRQoL.^4^ The increasing prevalence of TMDs can be attributed to their strong associations with stress, dietary habits and lifestyle factors.^5^ Notably, following the outbreak of the COVID‐19 pandemic, a significant increase in observed TMD symptoms has been observed, with a surge of over 60% compared to the previous year. This rise can be attributed to the psychological and environmental factors surrounding the pandemic.^5^

**TABLE 1:** Abbreviations used in the study.

Term	Abbreviation used
Biofeedback group	BG
Carleton University Responsiveness to Suggestion Scale	CURSS
Characteristic Pain Intensity	CPI
Cognitive behavioural therapy	CBT
Cognitive‐behavioural skills training	CBST
Control group	CG
Coping Strategies Questionnaire	CSQ
Center for Epidemiological Studies Depression scale	CES‐D
Graded Chronic Pain Score	GCPS
McGill Pain Questionnaire	MPQ
Multidimensional Pain Inventory	MPI
Oral Health Impact Profile	OHIP
Oral health related quality of life	OHRQoL
Pain disability index	PDI
Pain Stages of Change Questionnaire	PSCQ
Profile of Mood States	POMS
Research Diagnostic Criteria for temporomandibular disorders	RDC/TMD
Self‐care management	SCM
Temporomandibular disorders	TMDs
Temporomandibular joint	TMJ
Visual analog scale	VAS

The management of TMDs involves a multidisciplinary approach, including pharmacological interventions, physical therapy, dental treatments, and psychological interventions.^6^, ^7^, ^8^, ^9^, ^10^, ^11^ Among these, cognitive‐behavioural therapy (CBT) has gained recognition as an effective psychological intervention for managing chronic pain conditions, including TMDs.^9^, ^12^, ^13^ CBT aims to modify maladaptive thoughts, behaviours, and emotional responses associated with pain, thereby improving pain coping mechanisms and overall well‐being.^10^, ^14^

Standard treatment interventions for TMDs typically include non‐steroidal anti‐inflammatory drugs (NSAIDs), occlusal splints, physical therapy modalities, and patient education.^7^ While these treatments provide symptomatic relief, there is a growing interest in exploring the effectiveness of CBT as a complementary or alternative approach to standard treatments.^8^, ^15^, ^16^

Patients with TMD often seek medical attention in the advanced stages of the disease. In a follow‐up study, it was found that nearly 40% of patients who had discontinued initial conservative treatment without symptomatic improvement required subsequent treatment as the disease progressed to a chronic state.^6^ TMD affects the musculoskeletal system, including the neck and shoulders, and can be exacerbated by factors such as facial asymmetry and trismus.^9^ The treatment approach for TMD primarily involves conservative measures such as behaviour control, physical therapy, medication, and the use of occlusal stabilisation splints.^10^, ^11^, ^17^ However, the efficacy of these conservative treatments is limited due to the relatively brief education provided to patients during dental visits, where they are expected to manage their condition independently. Consequently, treatment outcomes vary significantly based on the patient's understanding and cooperation.^18^, ^19^, ^20^, ^21^, ^22^ Given the limitations of one‐time interventions, self‐directed treatment becomes crucial, with CBT, stress control, muscle relaxation, and posture correction showing effectiveness in addressing the underlying causes of TMD.^18^ Clinicians strive to restore normal function of the TMJ and alleviate pain and parafunctional activities burdening the TMJ. These objectives necessitate the adoption of self‐care management by the patients themselves.^19^ Self‐care management entails active patient involvement and adherence to recommended procedures.^20^, ^23^, ^24^

Hence, the present review was undertaken to answer the research question, ‘Is CBT as effective as standard intervention or a control group in the treatment of TMDs?’

## 2 MATERIALS AND METHODS

### 2.1 Eligibility criteria

**FIGURE 1:**
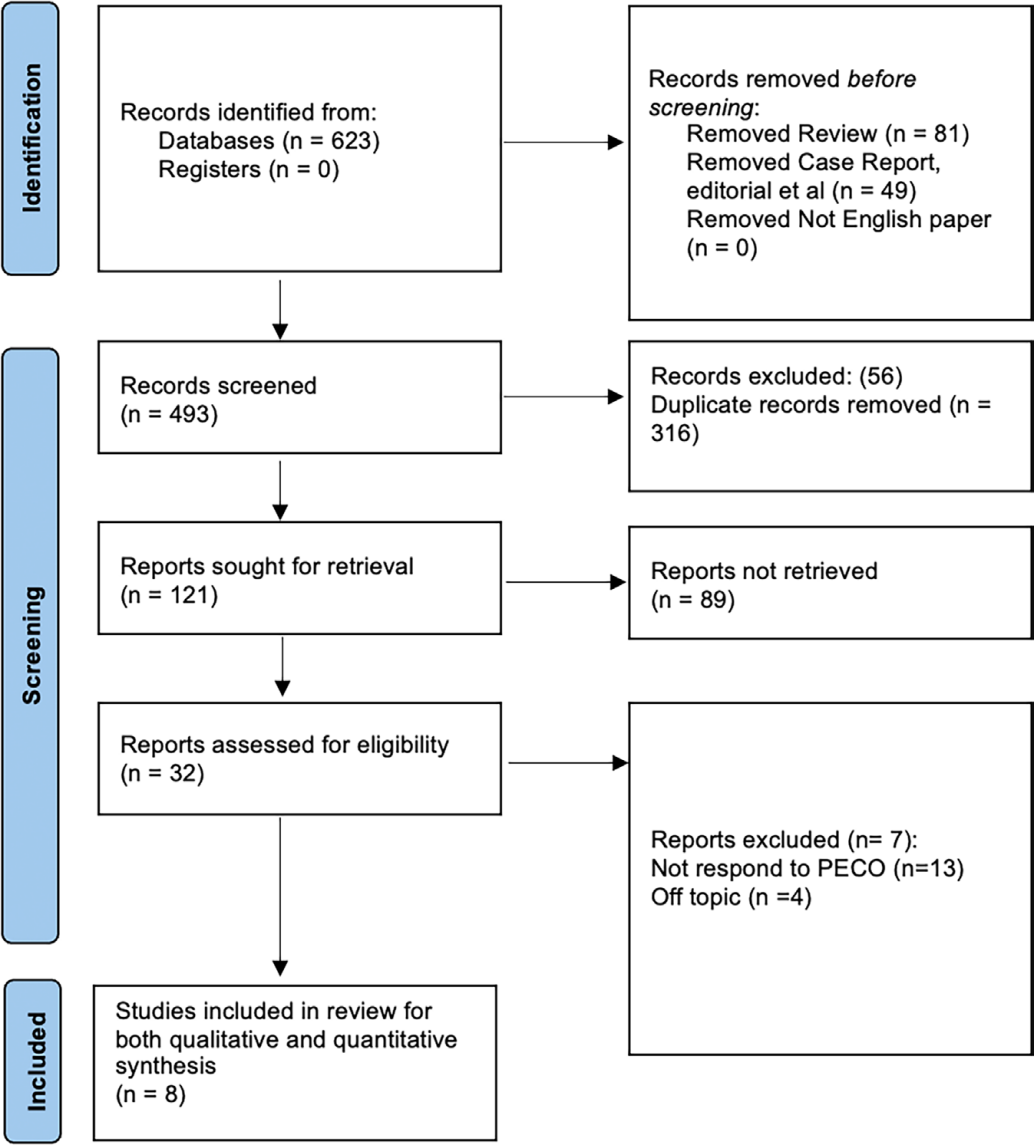
Graphical representation of the PRISMA guideline utilisation in the review.

The current systematic review adhered to the PRISMA guidelines^25^ to ensure a rigorous and transparent methodology (as seen in Figure 1). The review is registered with PROSPERO (CRD42023445847).

The PICOS model was employed to structure the research question and guide the systematic review process. The strategy ensured a comprehensive and focused approach to identifying relevant studies and extracting pertinent information.

Population (P): human individuals diagnosed with TMD.

Intervention (I) was CBT for TMD treatment either used alone or along with other treatment modalities.

Comparison (C): involved a group who received standard treatment approaches for TMD. Standard treatment approaches could include pharmacotherapy, physical therapy, splint therapy, or any other commonly employed interventions considered standard practise for managing TMD.

Outcome (O) assessed was effectiveness in TMD reduction. It included variables such as pain scores, jaw function, oral health‐related quality of life, mental well‐being, and other relevant outcome measures specific to TMD symptoms and treatment efficacy.

Study design (S) included clinical comparative studies.

Only RCTs, case–control studies, and comparative studies that evaluated the effectiveness of CBT compared to standard treatment or conventional interventions for TMD in a controlled setting were included, with no restriction on time periods. Case reports, editorials, reviews, and conference abstracts were excluded from the review to maintain methodological rigour and focus on higher‐quality evidence. Articles that did not compare CBT with standard treatment or conventional interventions were excluded from the review. Articles published in languages other than English were also not included.

### 2.2 Search strategy

The search strategy for this study, conducted various databases, employed Boolean operators and MeSH keywords to ensure a comprehensive and systematic approach to identifying relevant literature (as presented in Table 2). The search query was constructed using Boolean operators (AND, OR) to combine relevant terms and concepts. The MeSH (Medical Subject Headings) keywords were used to capture specific terms related to TMD and CBT. The search strategy aimed to identify studies that compared CBT to standard treatment in TMD. The search strategy included terms related to the population (temporomandibular disorders), intervention (cognitive‐behavioural therapy), comparison (standard treatment), and outcomes (e.g. pain, jaw function, quality of life). The MeSH keywords were used to ensure a comprehensive search, incorporating standardised medical terminology.

**TABLE 2:** Search protocol across different databases.

Database	Search terms
PubMed/MEDLINE	(temporomandibular disorders OR TMD OR TMJ disorder) AND (cognitive behavioral therapy OR CBT OR psychotherapy) AND (standard treatment OR conventional treatment OR traditional therapy)
Embase	(‘temporomandibular joint disorder’/exp OR ‘temporomandibular joint disorder’ OR ‘TMJ disorder’ OR ‘TMJ dysfunction’) AND (‘cognitive behavior therapy’/exp OR ‘cognitive behavior therapy’ OR ‘CBT’ OR ‘psychotherapy’) AND (‘standard treatment’ OR ‘conventional treatment’ OR ‘traditional therapy’)
PsycINFO	(TI = (temporomandibular disorder OR TMD OR TMJ disorder) OR AB = (temporomandibular disorder OR TMD OR TMJ disorder)) AND (TI = (cognitive behavioral therapy OR CBT OR psychotherapy) OR AB = (cognitive behavioral therapy OR CBT OR psychotherapy)) AND (TI = (standard treatment OR conventional treatment OR traditional therapy) OR AB = (standard treatment OR conventional treatment OR traditional therapy))
Scopus	TITLE‐ABS‐KEY(temporomandibular disorders OR TMD OR TMJ disorder) AND TITLE‐ABS‐KEY(cognitive behavioral therapy OR CBT OR psychotherapy) AND TITLE‐ABS‐KEY(standard treatment OR conventional treatment OR traditional therapy)
Web of Science	TS = (temporomandibular disorders OR TMD OR TMJ disorder) AND TS = (cognitive behavioral therapy OR CBT OR psychotherapy) AND TS = (standard treatment OR conventional treatment OR traditional therapy)
Cochrane Library	(Temporomandibular Disorders OR TMD OR TMJ Disorders OR TMJ Dysfunction) AND (Cognitive‐Behavioral Therapy OR Cognitive Therapy OR CBT) AND (Standard of Care OR Standard Treatment OR Conventional Treatment) AND (Pain Measurement OR Pain Assessment OR Pain Scores OR Visual Analog Scale) AND (Jaw Function OR Jaw Mobility) AND (Quality of Life OR Oral Health‐Related Quality of Life) AND (Randomized Controlled Trial OR Case–Control Studies OR Comparative Study)
CINAHL	(MH “Temporomandibular Joint Disorders”) AND (MH “Cognitive Behavioral Therapy”) AND (MH “Standard of Care”)
LILACS	(MH = “Temporomandibular Joint Disorders”) AND (MH = “Cognitive Behavioral Therapy”) AND (MH = “Standard of Care”)

### 2.3 Data extraction protocol

The data extraction process followed a systematic approach with detailed guidelines to maintain consistency and minimise potential bias. The reviewers underwent a rigorous training process to familiarise themselves with the data extraction protocol, including the specific variables to be extracted from each study. This training aimed to enhance their understanding of TMDs, CBT interventions and the outcomes of interest. The reviewers also had access to a comprehensive data extraction form specifically developed for this study. The data extraction form encompassed key information, such as study characteristics (e.g. study design, sample size, follow‐up period), participant characteristics (e.g. age, gender), intervention details (e.g. type of CBT, control treatment), outcome measures (e.g. pain assessment methods, jaw function measures, quality of life assessments), and relevant findings (e.g. changes in pain scores, improvements in jaw function). To ensure the accuracy and consistency of data extraction, the reviewers independently extracted data from the included studies. Any disagreements were resolved through discussions and referring to the study protocol.

### 2.4 Bias assessment

Bias assessment was conducted using the RoB 2.0 tool.^26^ This tool was employed to evaluate the methodological quality and potential biases in the included studies, ensuring a comprehensive assessment of the evidence.

## 3 RESULTS

### 3.1 Study characteristics

A total of 623 records were identified in the database. 493 of the articles were screened initially on the basis of their titles and abstract. 372 articles were excluded as they did not meet the predefined inclusion criteria and were irrelevant. 32 reports were selected for full screening. In the final phase, a total of eight clinical studies^27^, ^28^, ^29^, ^30^, ^31^, ^32^, ^33^, ^34^ that met the eligibility criteria for inclusion were included in this systematic review.

Table 3 provides a comprehensive overview of various studies conducted in different regions, focusing on demographic variables such as sample size, mean age, and gender ratio. Across the studies, there were variations in sample sizes, ranging from 21 to 148 participants. The mean age of the participants ranged from 25.7 to 39.4 years. The studies predominantly included female participants. The gender ratio varied across studies, with the majority of them having a higher proportion of female participants.

**TABLE 3:** Variables pertaining to the demographics of the included papers.

Study ID	Year	Region	Sample size (*n*)	Mean age (in years)	Gender ratio
Calderon et al.^27^	2011	Brazil	47	35.6	All females
Ferrando et al.^28^	2012	Spain	30	38.38 ± 16.57	21 females
Hwangbo et al.^29^	2023	South Korea	21	32.7 ± 13.2	16 females
Litt MD et al^30^	2010	USA	101	39.4 ± 12.1	85 females
Mishra et al^31^	2000	USA	94	35.76 ± 9.92	77 females
Shedden et al.^32^	2013	Germany	56	35.3 ± 13.94	44 females
Stam et al.^33^	1984	Canada	61	25.7 ± 7.0	51 females
Turner et al.^34^	2006	USA	148	37.3 ± 11.25	128 females

### 3.2 Main Findings

Table 4 provides an overview of TMD assessment methods, pain assessment methods, follow‐up periods, and the observed inferences. Each study investigated different protocols and groups, aiming to assess the effectiveness of various interventions on TMD symptoms. Except for the study of Stam et al.,^33^ all the other studies used RDC or TMD for TMD assessment.

**TABLE 4:** Variables pertaining to the TMD assessments and impact of CBT on TMD as observed in the included papers.

Study ID	Protocol	Groups assessed	TMD assessment method	Outcomes assessed	Follow‐up period	Inferences observed
Calderon et al.^27^	Case‐cohort	Amitriptyline (AMP), Amitriptyline+CBT, CBT + Placebo and CG	RDC/TMD	Pain (VAS), and OHIP	11 weeks	At the end of 11th week, VAS score was 38.7 in Group 3 (CBT with placebo) as compared to 52.6 in Group 4 (Placebo /Control), which was significant at *p* = .01. VAS scores of Group 3 was closer to Group 2 (38.7 versus 39.0). This indicates CBT may be as effective as a combination of AMP and CBT.
Ferrando et al.^28^	RCT	Hypnosis+CBT and conventional (splint therapy)	RDC/TMD	Pain (MPQ and MPI) Psychological distress	9 months	Pain variables in terms of frequency, intensity and severity was significantly reduced in CBT as compared to control group at *p* = .01, *p* = .00 and *p* = .00
Hwangbo et al.^29^	Case‐cohort	CBT (Behvioural therapy + physical therapy + drugs) and conventional treatment	RDC/TMD	Mouth opening, Tenderness in joint area, Pain and Stress	5.9 + 1.9 weeks	Mouth opening in experimental group was 3.5 mm, significant at *p* = .002 as compared to 1.0 mm in the control group (*p* = .072) after intervention. Pain intensity and joint sounds did not produce significant results.
Litt et al.^30^	RCT	Conventional (splint therapy + NSAIDs + soft diet) and CBT + conventional	Questionnaire and RDC/TMD	Pain measured by MPI; Depression by CES – D and Coping by PSCQ	14 days post treatment	Patients in the CBT+ conventional therapy group experienced steeper decrease in pain and coping changes over time as compared to standard therapy alone. Additionally, scores for somatization and desire to receive therapy were statistically lower in the standard treatment group.
Mishra et al.^31^	Case‐cohort	BG, CBST, BG + CGST and CG	Questionnaire and RDC/TMD	Chronic Pain assessed by GCPS and Various emotions by POMS	8 weeks after treatment in all intervention groups and 3 months later in the CG	With the exception of the CG all the groups showed significant improvement in terms of pain. The reduction in pain intensity in CBST was 31.8% as compared to 28% of CG while BG showed 52.2%. BG group showed superior results than the others.
Shedden et al.^32^	RCT	BG+ CBT and splint therapy	RDC/TMD	Pain assessed by PDI; Jaw use limitations; Coping and emotional well being	6 months	While pain intensity and disability showed similar results in improvement. Pain coping skills in BG + CBT group was greater.
Stam et al.^33^	Case‐cohort	Hypnosis+CBT, relaxation+CBT and CG	Assessment based on clinical examination	Pain intensity measured by CURSS and TMD symptoms such as limitation of jaw mobility and joint sounds	2–4 weeks in the intervention groups and 4–6 weeks in the CG	The reduction in pain scores was not significant at *p* = 1.49 between the two intervention groups, but was definitely significant with CG at *p* < .01. This suggests that CBT combined either with hypnosis or relaxation fair significantly better than the control group
Turner et al.^34^	RCT	CBT and SCM	RDC/TMD	GCPS‐based questionnaire	8 weeks	The CBT group saw changes that were noticeably greater than those in the SCM group throughout the follow‐up period in terms of frequency of pain, jaw function, and the depression‐related symptoms.

The CBT group of Calderon et al.^27^ were subjected to a protocol of expository lectures, relaxation training, and coping strategies for 90 min over a 7‐week period. In the study of Ferrando et al.,^28^ the experimental group was subjected to six sessions. The protocol included hypnosis training and cognitive restructuring by reducing the pain interface. Hwangbo et al.^29^ used a smartphone app that identified pain and inputted pain levels at various time intervals. The pain score was marked on a visual analogue scale, which allowed the patients to subjectively analyse the score. This helped the patients identify the kind of pain. The app facilitated patients to be aware of TMD pain so as to induce a voluntary avoidance and mouth restriction activity to provide therapeutic effect. Litt MD et al.^30^ devised a CBT programme that combined relaxation exercises, stress management, and cognitive restructuring. The CBT group of Mishra et al.^31^ included relaxation training, application of distraction methods to control pain, scheduling of pleasant activities, self‐instruction training, and training of social skills. Shedden et al.^32^ educated patients regarding the psychosocial aspect of pain and trained them in habit reversal techniques so as to adopt a relaxed jaw posture. Stam et al.^33^ used the hypnosis technique for CBT. Turner et al.^34^ included relaxation training and breathing exercises. A discussion was held on fear avoidance, identification and challenging thoughts related to pain.

Overall, seven studies^27^, ^28^, ^29^, ^30^, ^32^, ^33^, ^34^ showed that CBT was better or showed greater efficacy in reducing TMDs as compared to conventional treatment or a control group. The only study that showed almost no difference between CBT and CG was that of Mishra et al.^31^ But all groups in this study showed significant differences between pre‐ and post‐intervention. In all studies, pain scores were significantly high.

### 3.3 Risk of bias assessment

**FIGURE 2:**
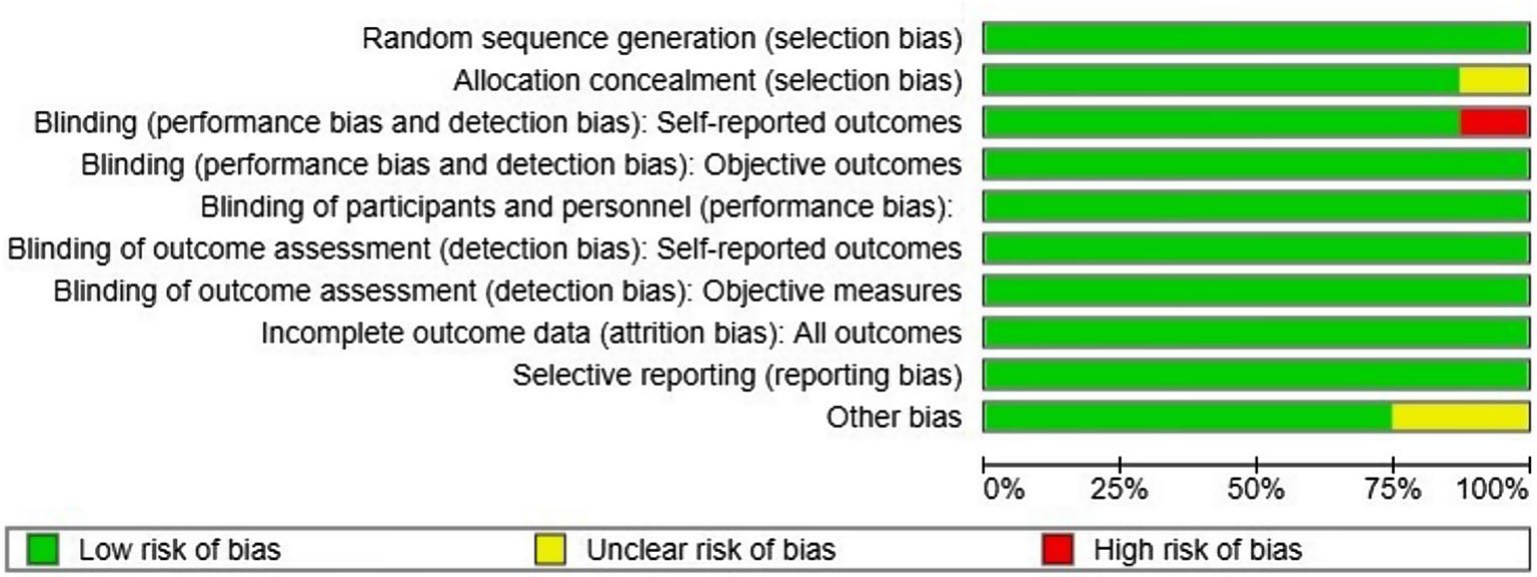
Risk of bias graph of included studies.

**FIGURE 3:**
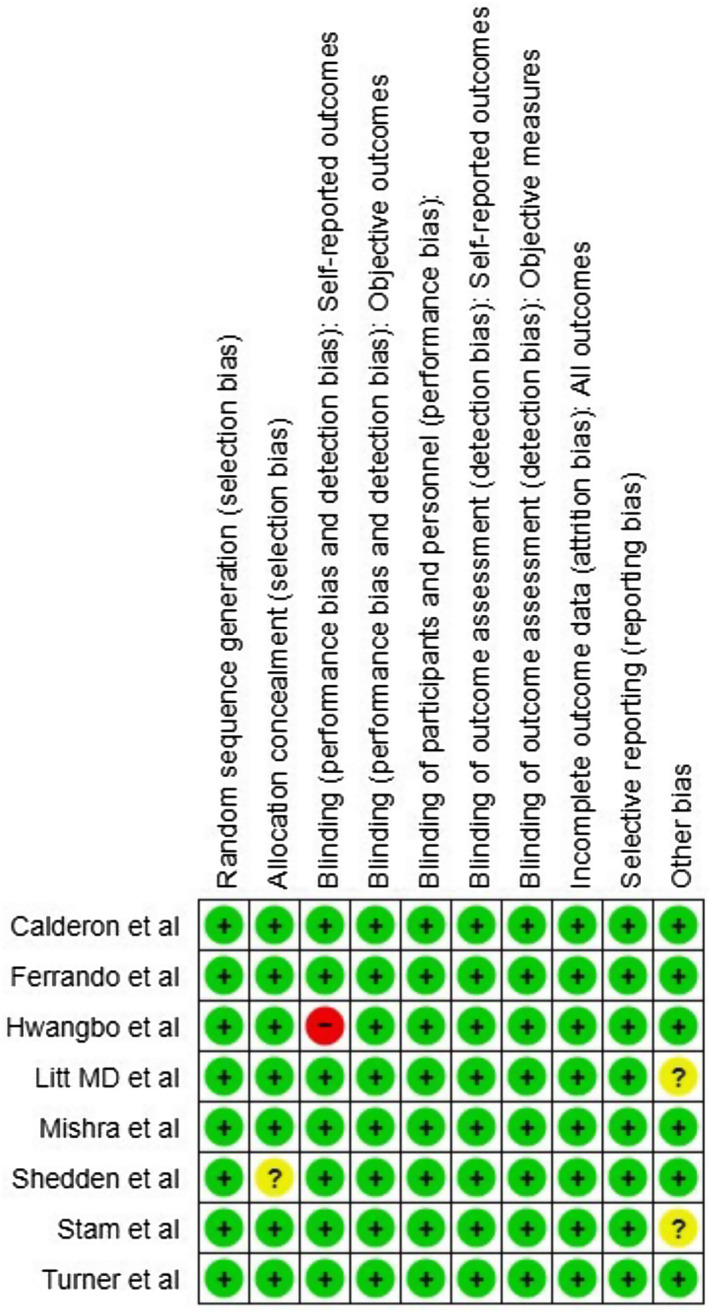
Risk of bias summary of included studies.

Overall, RoB‐2 shows a low risk of bias in all studies as seen in Figure 2. The study of Shedden et al.^32^ was marked by uncertain bias in the domain of selection bias as it did not have a true control or placebo group. Detection bias in Hwangbo et al^29^ was marked as high risk as mobile phone applications were used for delivering behavioural therapy in the experimental group and to detect pain. The studies of Litt et al.^30^ (14 days) and Stam et al.^33^ (2–4 weeks) were marked as having unclear bias in the other risk category, as both studies reported clinical findings immediately after the treatment period (Figure 3).

## 4 DISCUSSION

The significance of this review is multi‐fold. At the foremost, these studies contribute to the scientific understanding of TMD, a common condition characterised by jaw pain, dysfunction, and associated symptoms. By investigating different assessment methods, pain measurement techniques, and interventions, these studies enhance our knowledge of TMD's aetiology, symptomatology, and potential treatment options. Furthermore, the findings of these studies have clinical implications.^35^, ^36^ They shed light on the effectiveness of interventions such as CBT, self‐administered treatment, hypnosis, and combinations of therapies in managing TMD symptoms. The observed reductions in pain scores, improved jaw function, and enhanced mental well‐being in response to these interventions offer potential treatment strategies for healthcare professionals, including dentists, orofacial pain specialists and therapists specialising in TMD. Moreover, these studies contribute to the development of evidence‐based guidelines and treatment protocols for TMD management. The findings of this review emphasise the potential benefits of combining therapies, such as CBT with conventional treatments or behavioural group therapy, to achieve optimal outcomes.^37^, ^38^ This recognition of the multifaceted nature of TMD and the importance of addressing both physical and psychological factors in its management contributes to a more holistic approach to patient care.

In terms of pain reduction, several studies reported positive outcomes in this review. Calderon et al.^27^ found that women in the amitriptyline+CBT group experienced reduced pain scores, while Mishra et al.^31^ reported significant improvements in pain across most intervention groups. Additionally, Litt et al.^30^ and Shedden et al.^32^ all found that interventions combining CBT with conventional therapy or behavioural group therapy led to greater improvements in pain frequency compared to standard therapy or splint therapy alone. Regarding mental well‐being, Ferrando et al.^28^ reported decreased mental distress in patients receiving CBT interventions. Calderon et al.^27^ observed a reduction in overall depressive symptoms in the amitriptyline+CBT group. These findings suggest that CBT‐based interventions may have a positive impact on the psychological aspects of TMD. However, heterogeneity in the specific interventions and assessment methods employed across the studies cannot be overlooked. For example, Hwangbo et al.^29^ reported greater improvement in TMD‐related pain and joint clicking sounds with self‐administered treatment, while Stam et al.^33^ found that hypnosis+CBT led to reduced pain scores. Further differences may be attributed to variations in the study protocols, sample sizes, and assessment tools employed.

Most of the studies exploring the effectiveness of combined CBT interventions have classified TMD cases into distinct categories based on the presence and severity of comorbid mental illnesses.^30^, ^34^, ^39^, ^40^, ^41^, ^42^, ^43^, ^44^ One paper highlighted the effectiveness of a combined intervention involving CBT, intraoral appliances, stress management, and biofeedback for TMD pain.^45^ These findings collectively underscore the importance of considering a comprehensive treatment approach that incorporates CBT for patients presenting with TMD and concurrent psychosocial issues. In addition, several investigations have examined the effects of CBT on different types of orofacial pain conditions associated with TMD.^29^, ^46^, ^47^, ^48^ One study investigated the effectiveness of CBT specifically for patients with unexplained chronic orofacial pain.^49^ However, their study did not meet the inclusion criteria for our systematic review as it did not utilise a case–control design.

CBT encourages individuals to identify and challenge negative thought patterns related to stress. By reframing irrational thoughts and focusing on more realistic and positive thinking, CBT can reduce the emotional impact of stress. Stress is widely recognised as a common trigger for TMD symptoms. CBT helps individuals identify stressors and develop strategies to manage and reduce stress effectively. TMD pain can lead to catastrophizing thoughts, where individuals anticipate severe pain and feel helpless. CBT assists in altering these thought patterns by promoting a more adaptive way of perceiving and coping with pain. CBT equips individuals with a range of pain coping strategies, such as relaxation techniques, mindfulness, and guided imagery. These strategies help TMD patients manage pain more effectively, reducing its impact on their daily lives.

The majority of the studies in the current review evaluated the efficacy of CBT in combination with other therapy modalities. However, if there were more studies to investigate the solitary treatment approach of CBT, the results may potentially yield a clearer understanding of its true efficacy in managing TMD. By isolating CBT as a standalone treatment, researchers would be able to gauge its effectiveness independent of potential confounding variables introduced by the concurrent use of multiple therapeutic methods. This focused approach could offer valuable insights into the unique benefits that CBT may bring to individuals with TMD and may help in determining whether it should be recommended as a primary treatment modality. Further research with this specific focus could contribute significantly to the refinement of treatment guidelines for TMD patients.

This review is not without its limitations, which should be taken into account when interpreting the findings. Firstly, the heterogeneity in study design, sample size, and assessment methods across the studies introduces potential sources of bias and variability. Differences in protocol, such as the duration and intensity of interventions, follow‐up periods, and outcome measures, make it challenging to directly compare the results and generalise the findings to broader populations. Another limitation is the potential for selection bias. The studies often recruited participants from specific clinical settings or targeted certain patient groups, which may not fully represent the diverse population with TMD. Consequently, the findings may not be applicable to all individuals with TMD or those from different demographic backgrounds. Also, the reliance on self‐reported measures introduces the possibility of subjective reporting bias. Pain and symptom assessments primarily relied on questionnaires, pain rating scales, and oral health‐related quality of life measures.^50^, ^51^, ^52^ These subjective measures can be influenced by individual interpretation, recall bias, or social desirability bias, potentially affecting the accuracy and reliability of the reported outcomes. Additionally, the relatively short follow‐up periods in some studies limit the understanding of the long‐term effectiveness and sustainability of the interventions. TMD is a chronic condition that may require extended treatment and monitoring, and longer follow‐up periods would provide more comprehensive insights into the durability of the observed improvements.

## 5 CONCLUSION

The collective findings from the studies under assessment in this review provide valuable insights into the management of this prevalent condition. The studies have contributed to our scientific understanding of TMD by elucidating its aetiology, symptomatology, and potential treatment options. The observed reductions in pain scores, improvements in jaw function, and enhanced mental well‐being highlight the potential benefits of interventions such as CBT, self‐administered treatment, and combinations of therapies in alleviating TMD symptoms. These findings have important clinical implications for healthcare professionals involved in the care of TMD patients, offering evidence‐based strategies to enhance treatment outcomes and improve patients' OHRQoL. However, it is crucial to acknowledge the limitations present in these studies. Therefore, to address these limitations, future research should strive for standardised protocols, larger and more diverse samples, objective outcome measures, and longer‐term follow‐up. This would enhance the validity, reliability, and generalizability of the findings, leading to more robust evidence for the optimal management of TMD.

## AUTHOR CONTRIBUTIONS

Sahana Shivakumar: writing—original draft preparation, writing review and editing, supervision. Nishath Sayed Abdul: Conceptualization, software, validation, formal analysis. Bhuvan Jyoti: Conceptualization, methodology, software, validation, formal analysis, investigation; data curation, writing—original draft preparation. Veena Kalburgi: investigation; data curation. Giuseppe Minervini: writing—review and editing, supervision. Marco Cicciù: writing—review and editing, supervision. All authors have read and agreed to the published version of the manuscript.

## FUNDING INFORMATION

This research received no external funding.

## CONFLICT OF INTEREST STATEMENT

The authors declare no conflict of interest.

## INSTITUTIONAL REVIEW BOARD STATEMENT

Not applicable.

## Data Availability

All data described in the study are presented in the manuscript. The datasets analysed are available from the corresponding author on reasonable request.
